# Book Reviews

**Published:** 1873-07-01

**Authors:** 


### Book Reviews.

Hand-book for the Physiological Laboratory. By E. Klein, M.D., J. Burton Sanderson, M.D., Michael Foster, A.M., M.D., and F. Louder Brunton, M.D. Edited by J. Burton Sanderson. In two volumes, with one hundred and thirty-three plates. Vol. I., Text; Vol. II., Plates. Philadelphia: Lindsay & Blakiston, 1873.

This work, according to the author's preface, is intended for beginners in physiological work. It is a book of methods, not a compendium of the science of physiology, and consequently claims a place rather in the laboratory than in the study. The practical object of the book is kept strictly in view, both in the arrangement and the selection of the subjects. The plates are excellent, comprising some three hundred and fifty illustrations.

The names and reputation of the several gentlemen by whom the different portions of the work have been prepared are sufficient guarantee of the practical value and importance of the matter contained.

A Treatise on the Principles and Practice of Medicine. By Austin Flint, M.D. Fourth Edition. Carefully Revised. Philadelpnia: Henry C. Lea. 1873.

The scope and character of this standard work are too generally known to require any extended review. Ever since the first edition was placed before the profession, in 1868, it has continued to rank as the leading American text-book on the practice of medicine. The present edition has been carefully revised and brought fully up to the times in all departments.

